# Supplementary material for: A non-Newtonian fluid quasi-solid electrolyte designed for long life and high safety Li-O2 batteries
Source: Nat Commun. 2023 Apr 20;14:2268. doi: 10.1038/s41467-023-37998-5 (PMC10119181; doi:10.1038/s41467-023-37998-5)
Supplement: Supplementary file 1 — Supplementary Information [file 41467_2023_37998_MOESM1_ESM.pdf]

Supplementary Information

A non-Newtonian fluid quasi-solid electrolyte designed for long life and high safety Li-O_2 batteries

*Guangli Zheng [1], Tong Yan [1], Yifeng Hong [2], Xiaona Zhang [1], Jianying Wu [2], Zhenxing Liang [1], Zhiming Cui [1], Li Du [1], Huiyu Song [1,] **

[1]Guangdong Provincial Key Laboratory of Fuel Cell Technology, School of Chemistry and Chemical Engineering, South China University of Technology, Guangzhou 510641, China

[2]Department of Civil Engineering, South China University of Technology, Guangzhou 510641, China

[*]Email: hysong@scut.edu.cn

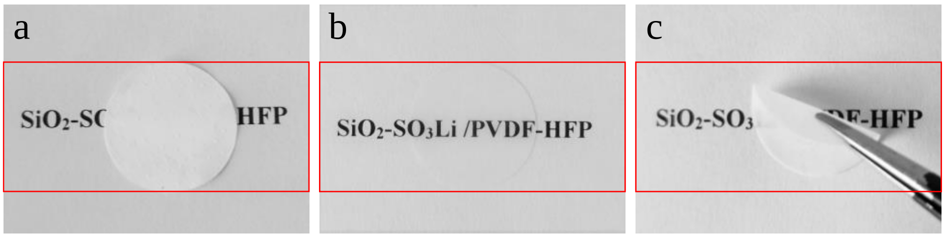

Before dipping After dipping Bend

Supplementary Fig. 1 Digital photos of SiO_2-SO_3Li/PVDF-HFP a before and **b** after dipping in liquid electrolyte, and it turns transparent after absorbing liquid electrolyte; **c** It remains flexible after bending several times.

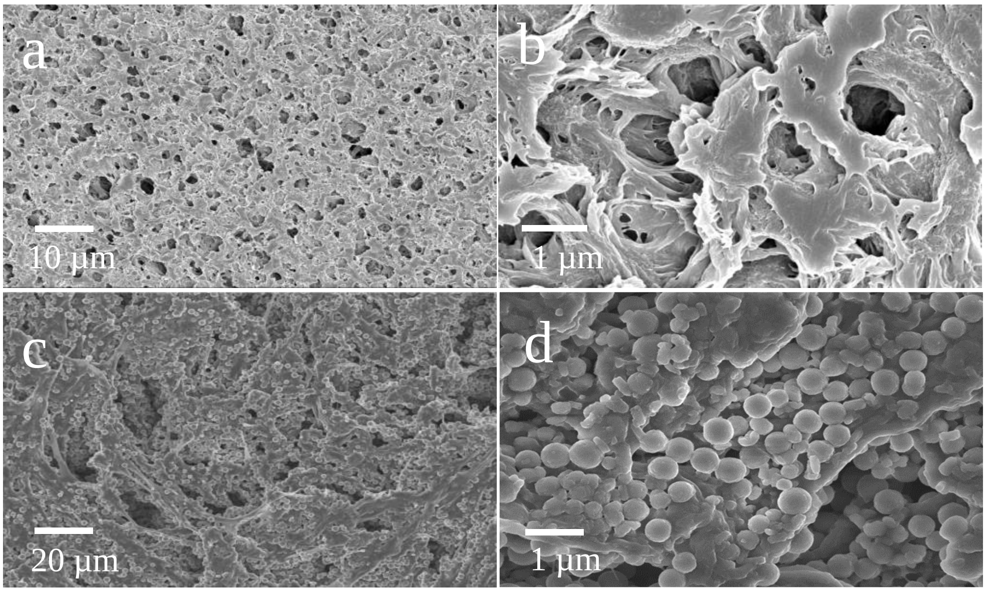

Supplementary Fig. 2 SEM analysis. **a-b** PVDF-HFP and **c-d** SiO_2/PVDF-HFP.

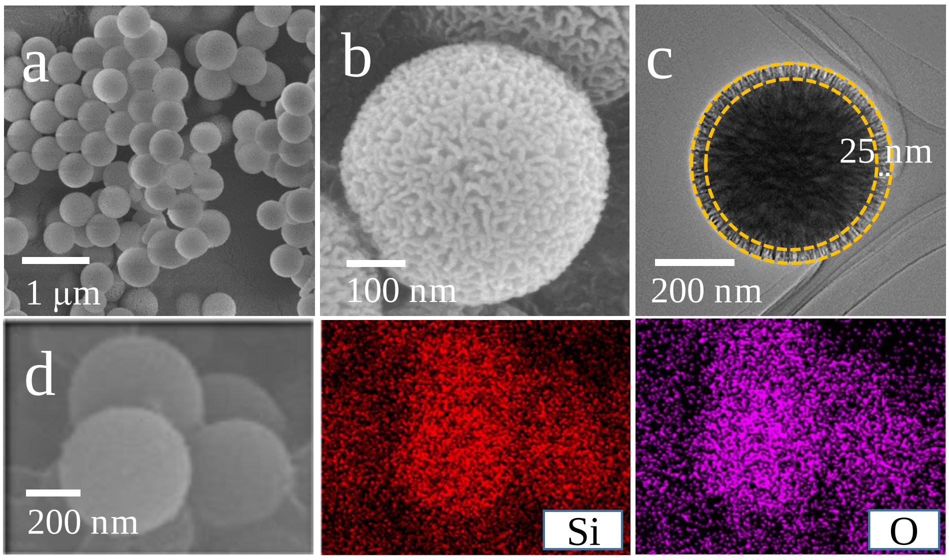

Supplementary Fig. 3 Characterization of SiO_2 particles. a-b SEM analysis; **c** TEM images; **d** EDS elemental mapping.

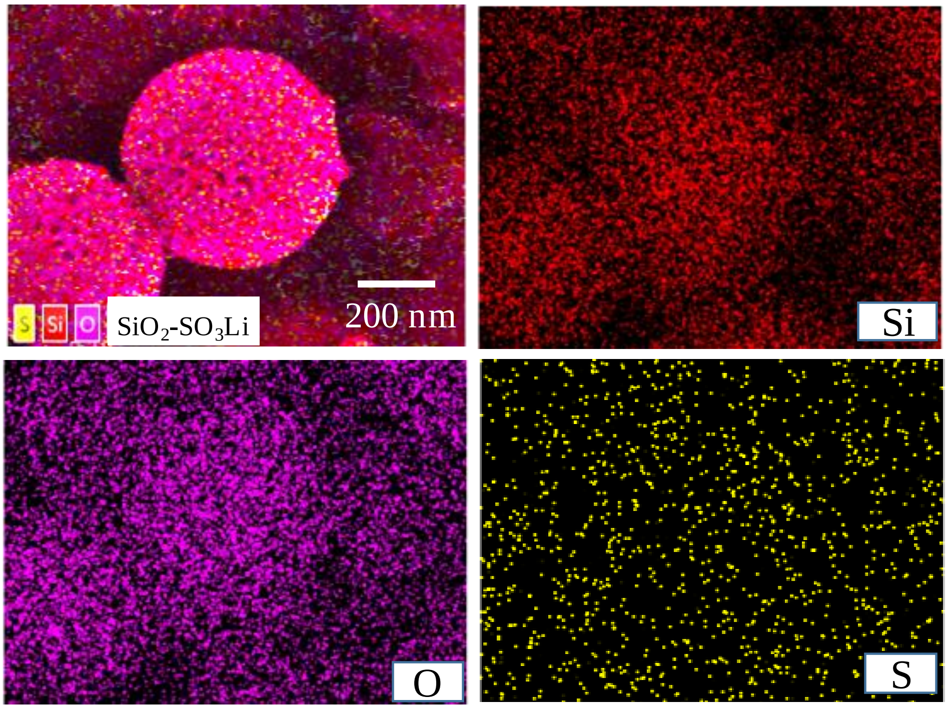

Supplementary Fig. 4 EDS elemental mapping of SiO_2-SO_3Li particles.

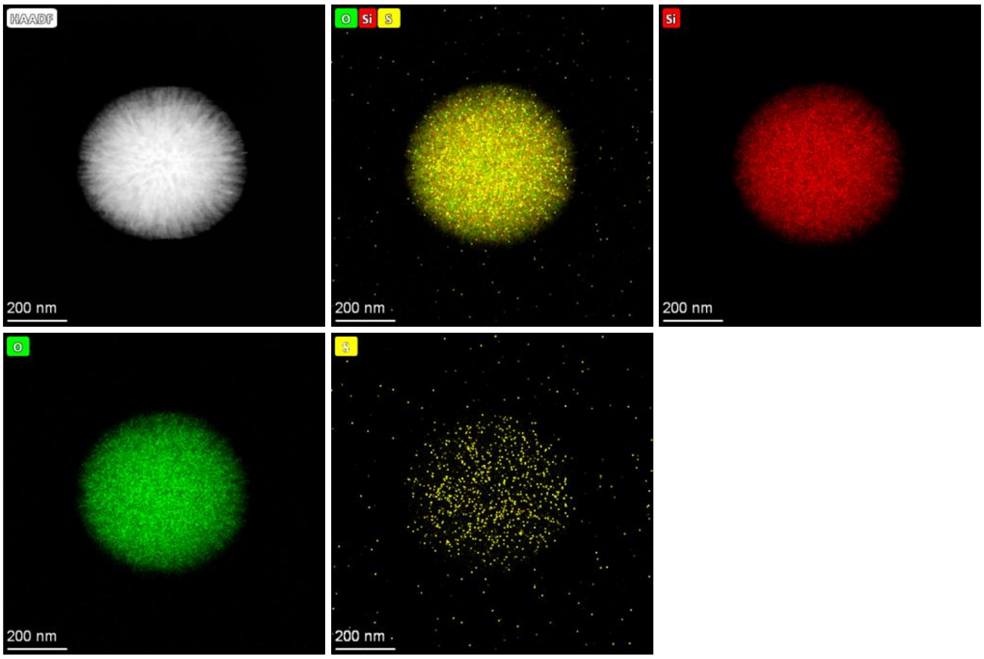

Supplementary Fig. 5 HADDF-STEM images and the corresponding elemental mapping of SiO_2-SO_3Li particles.

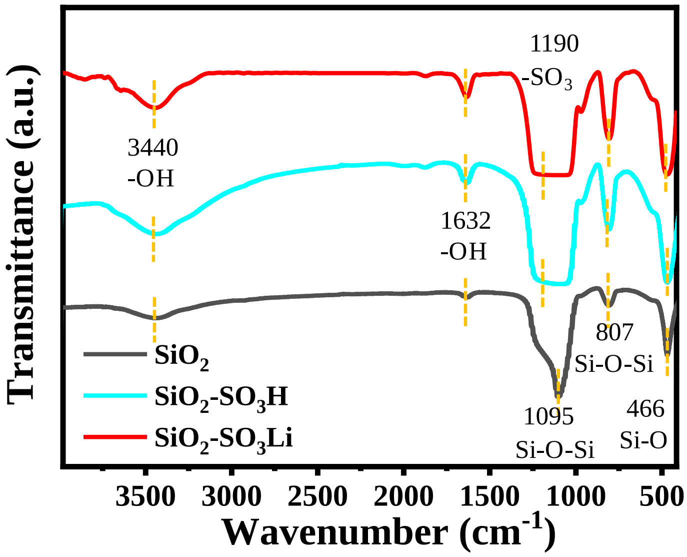

Supplementary Fig. 6 FTIR spectra of porous SiO_2, SiO_2-SO_3H and SiO_2-SO_3Li nanoparticles.

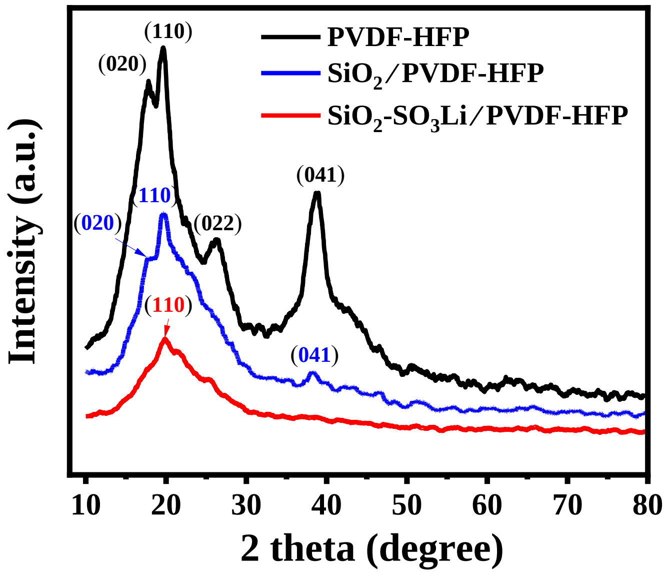

Supplementary Fig. 7 XRD analysis

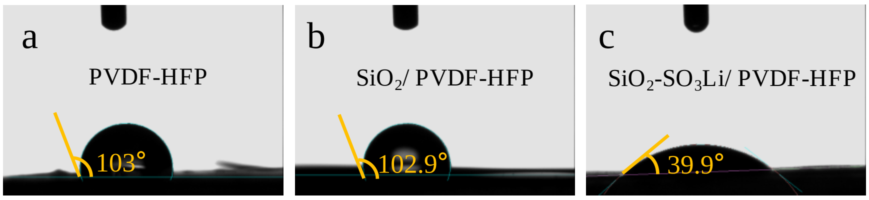

Supplementary Fig. 8. Contact angle between polymer film and liquid electrolyte (1 M LiTFSI/TEGDME): a PVDF-HFP; **b** SiO_2/PVDF-HFP; **c** SiO_2-SO_3Li/PVDF-HFP.

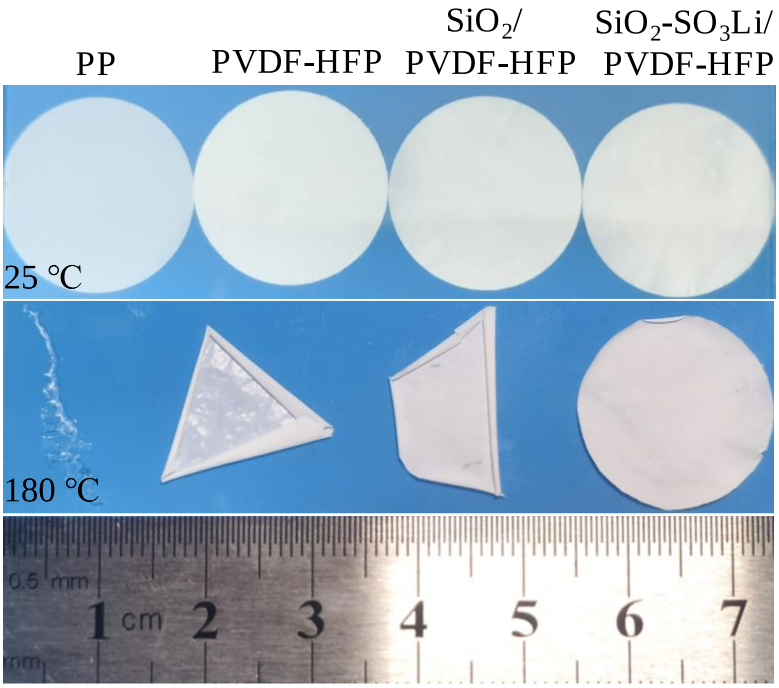

Supplementary Fig. 9. Digital photos before and after heating at 180 ℃ for 1 h.

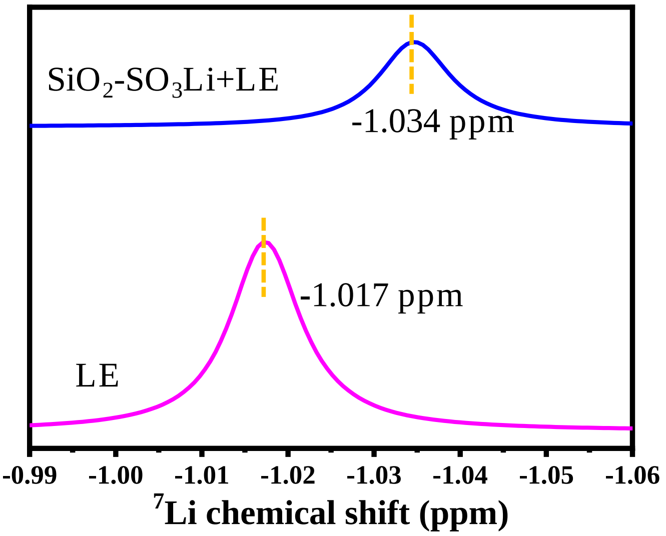

Supplementary Fig. 10. NMR analysis of ^{7}Li chemical shift. The rose line represents liquid electrolyte (LE) and blue line represents SiO_2-SO_3Li dipped in LE.

Supplementary Note 1.

The high Li^+ conductivity of quasi-solid electrolyte SiO_2-SO_3Li/PVDF-HFP at room temperature can be mainly attributed to the plenty of liquid electrolyte LiTFSI/TEGDME inside and Li^+-conductive groups -SO_3Li.

1) The electrolyte uptake of SiO_2-SO_3Li/PVDF-HFP reaches 380 wt% the dead weight, which is dominant the Li^+ conductivity.
2) Previous reports demonstrated that the sulfonate groups -SO_3Li with electronegativity can simultaneously facilitate access by the electrolyte to promote ion pair dissociation and increase the mobility of Li^+ [1]. Furthermore, -SO_3Li sites also provide the lithium ion hopping pathways to improve Li^+ conductivity[2,3].
3) The polymer skeleton PVDF-HFP provides a certain amount of Li^+ driving force through its segmental motion.

Therefore, the NNFQSE SiO_2-SO_3Li/PVDF-HFP with semi-dry surface can be seen as a "liquid electrolyte" to some extent, which exhibits high ion conductivity 5.4×10^{-3} S/cm at room temperature, equivalent to the reported conventional liquid electrolyte LiTFSI/TEGDME[4].

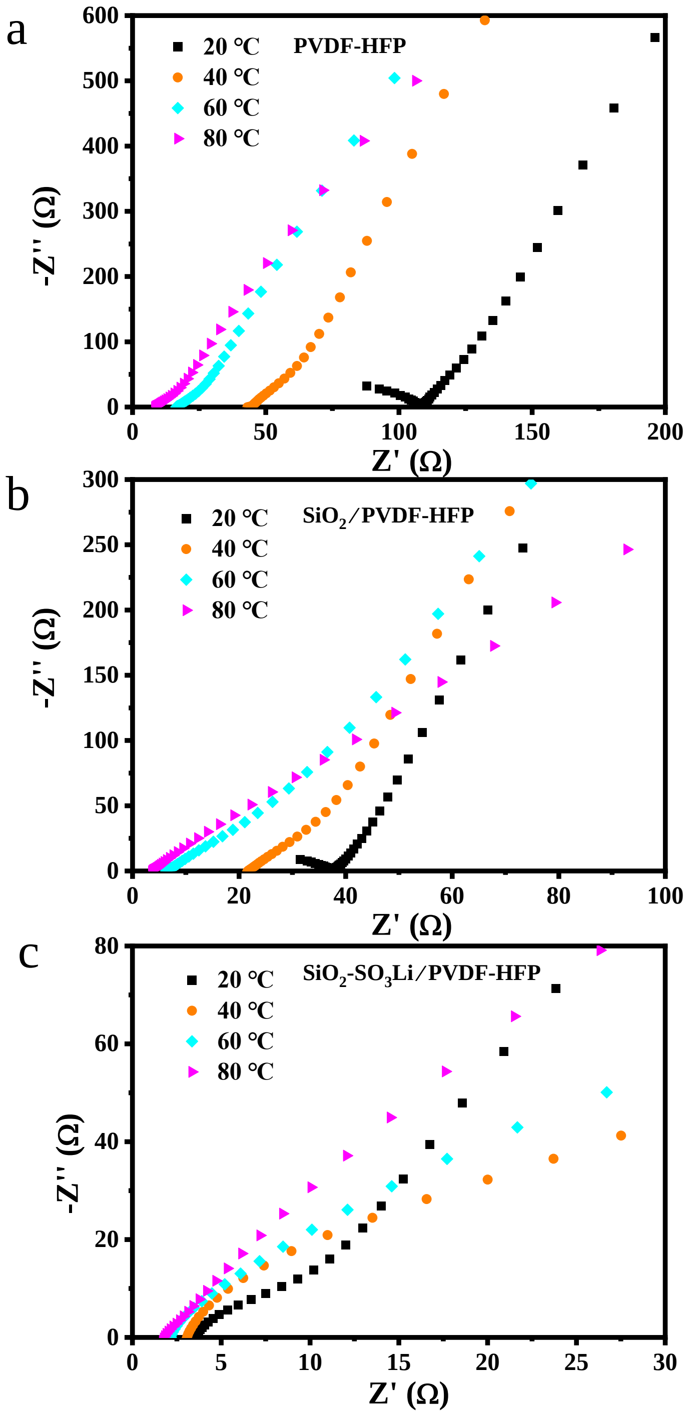

Supplementary Fig. 11 EIS Nyquist plots at different temperatures.

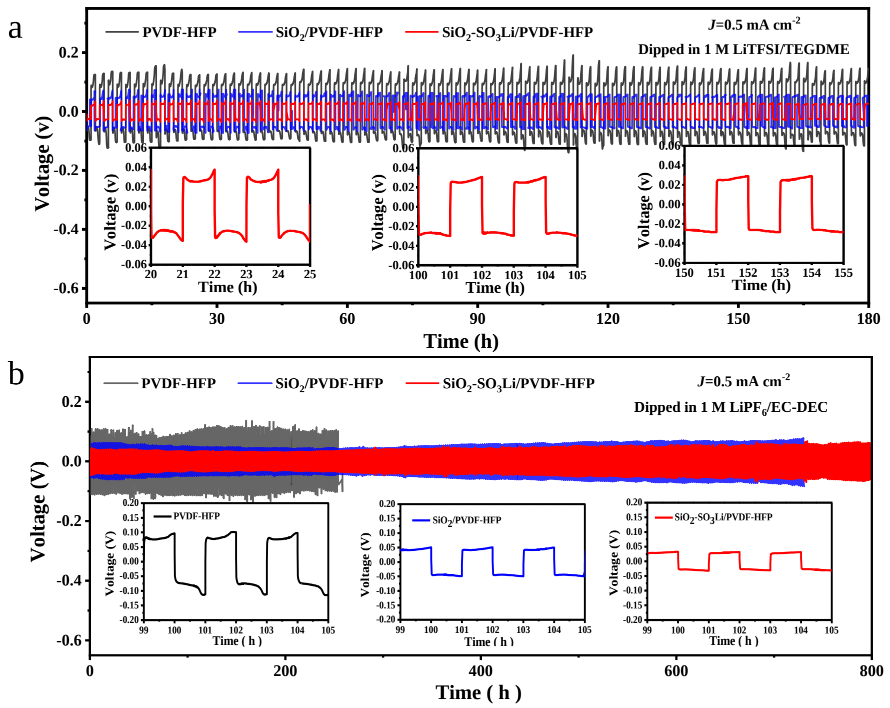

Supplementary Fig. 12 Symmetric lithium batteries with quasi-solid electrolytes at a current density of 0.5 mA cm^{-2} at 25 °C. **a** Dipped in 1 mol L^{-1} LiTFSI/TEGDME, and the inserts are selected voltage profiles for different time; **b** Dipped in 1 mol L^{-1} $LiPF_6$/EC-DEC, and the inserts are three kinds of voltage profiles at the same time for comparison.

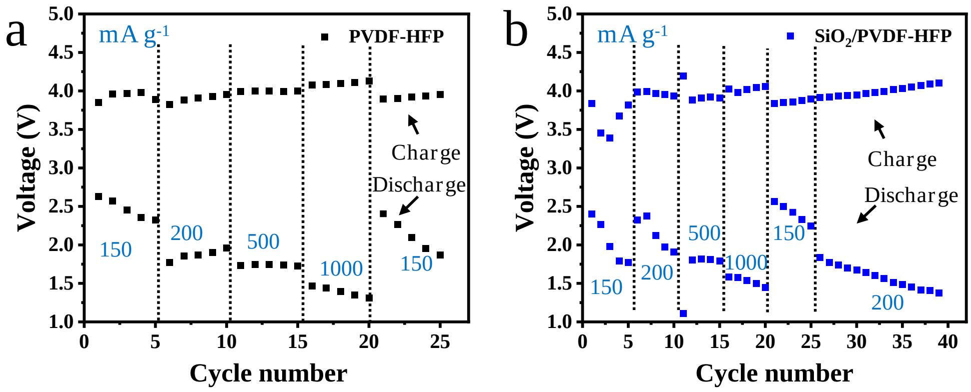

Supplementary Fig. 13 Rate performances of Li-O_2 cells with **a** PVDF-HFP and **b** SiO_2/PVDF-HFP.

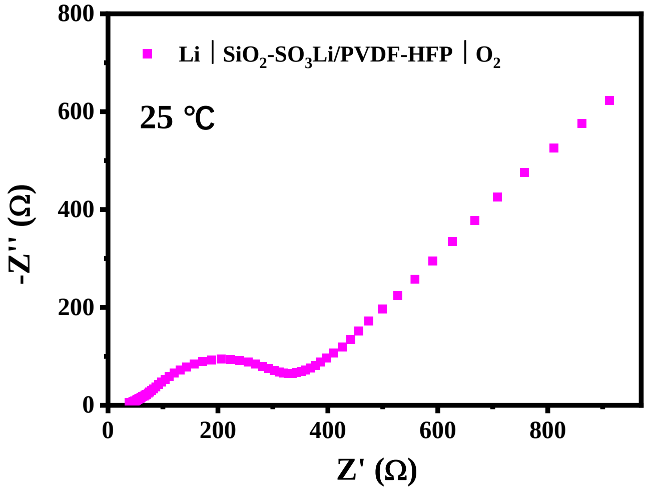

Supplementary Fig. 14 EIS curve of Li | SiO_2-SO_3Li /PVDF-HFP | O_2 after cycling.

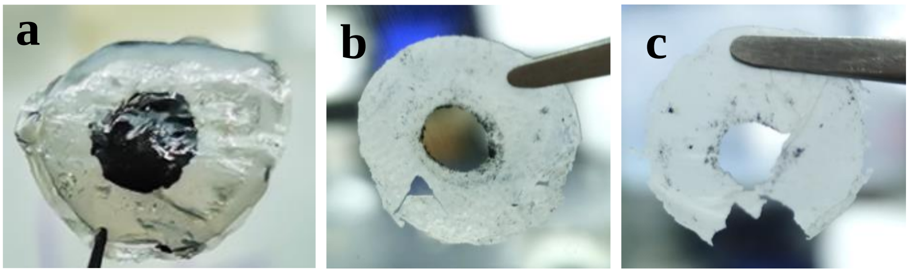

Supplementary Fig. 15 Digital photos after cycling. **a** SiO_2-SO_3Li/PVDF-HFP; **b** SiO_2/PVDF-HFP; **c** PVDF-HFP.

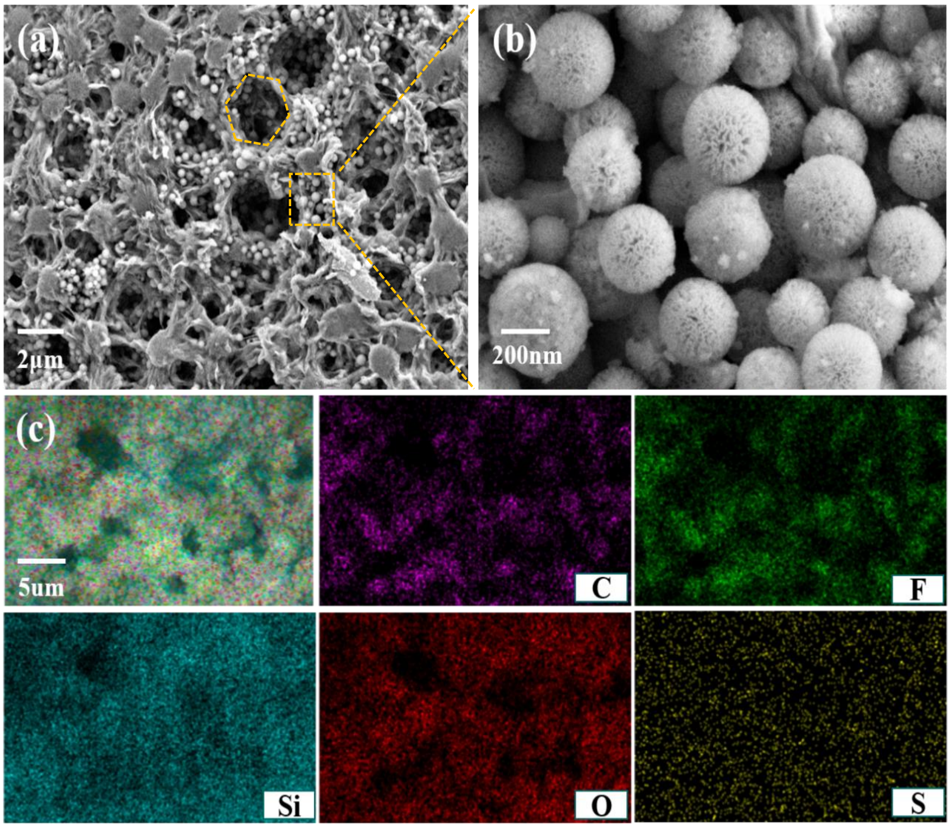

Supplementary Fig. 16 The bird view of SEM analysis and EDS elemental mapping of SiO_2-SO_3Li/PVDF-HFP after 5000 h Li-O_2 cycling.

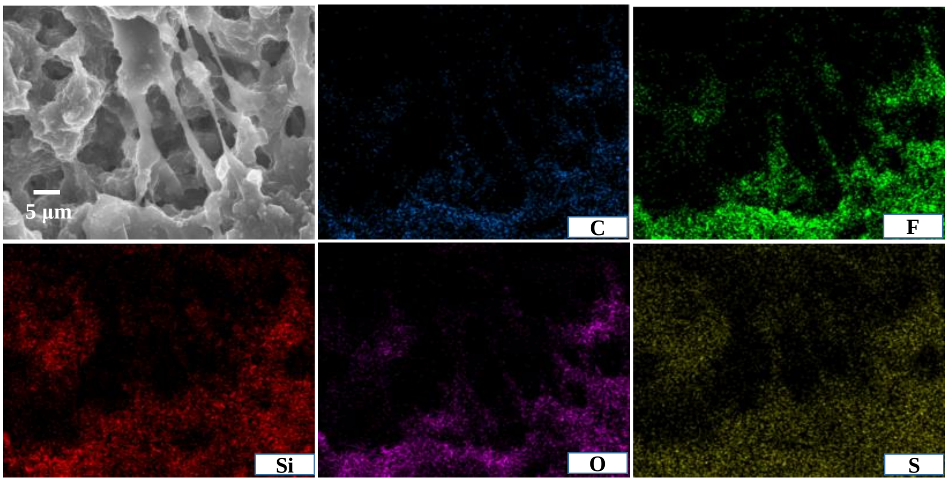

Supplementary Fig. 17 Cross section EDS elemental mapping of SiO_2-SO_3Li /PVDF-HFP after 5000 h Li-O_2 cycling.

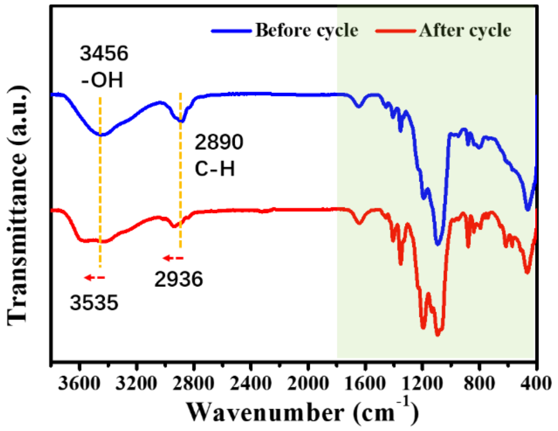

Supplementary Fig. 18 FTIR spectra of NNFQSE SiO_2-SO_3Li/PVDF-HFP before and after 5000 h cycling.

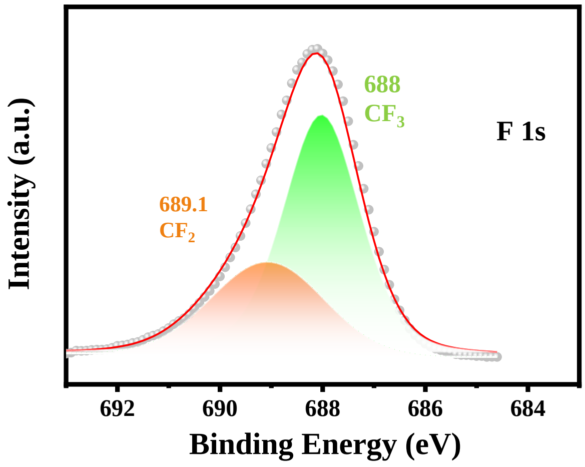

Supplementary Fig. 19 F 1s XPS analysis of NNFQSE SiO_2-SO_3Li/PVDF-HFP before absorbing liquid electrolyte.

Supplementary Note 2.

After long-term cycling, the lithium anode surface of Li｜SiO_2-SO_3Li/PVDF-HFP｜O_2 exhibits bright with metallic luster (Supplementary Fig. 20a), and its SEM morphology shows smooth without obvious dendrite growth (Supplementary Fig. 20d-e). While the anode of Li｜SiO_2/PVDF-HFP｜O_2 turns black (Supplementary Fig. 20b) and appears rough surface (Supplementary Fig. 20f-g). And the lithium anode of Li｜PVDF-HFP｜O_2 turns pulverized (Supplementary Fig. 20c).

For cathode, in order to better understand how the SiO_2-SO_3Li/PVDF-HFP electrolyte affects the growth and decomposition of discharge products, we investigated the morphology and crystal structure of the cathode after discharge and charge. As shown in Supplementary Fig. 23, high-magnification SEM images reveal that the discharge products of Li_2O_2 are formed on the cathode (Supplementary Fig. 23a-b) and completely disappeared after charging (Supplementary Fig. 23c-d), demonstrating good reversibility during the charge/discharge process. Moreover, X-ray diffraction (XRD) analyses of the pristine, discharged, and charged cathodes were carried out in Supplementary Fig. 23e. The discharged cathode exhibits two weak peaks located at 32.9° and 35.8°, which correspond to the (100) and (101) crystal surfaces of Li_2O_2, respectively[5]. After charging, the peaks completely disappear and recover to be consistent with the pristine crystal structure. These results perfectly validate the SiO_2-SO_3Li/PVDF-HFP electrolyte exhibits robust chemical stability against products during cycling[6].

In addition, after 5000 h cycling, the cathode of Li｜SiO_2-SO_3Li/PVDF-HFP｜O_2 still maintains hollow tubular structure and there only exists little residue of solid-state products (Supplementary Fig. 24a). In contrast, the cathode structure of control groups becomes deformation or even collapsed, and there also filled with residue, which hinders the persistent cycling (Supplementary Fig. 24b-c). These results further

confirm that the NNFQSE SiO_2-SO_3Li/PVDF-HFP can trap the liquid electrolyte inside to prevent the side reaction with cathode and reduce the residue of solid-state products.

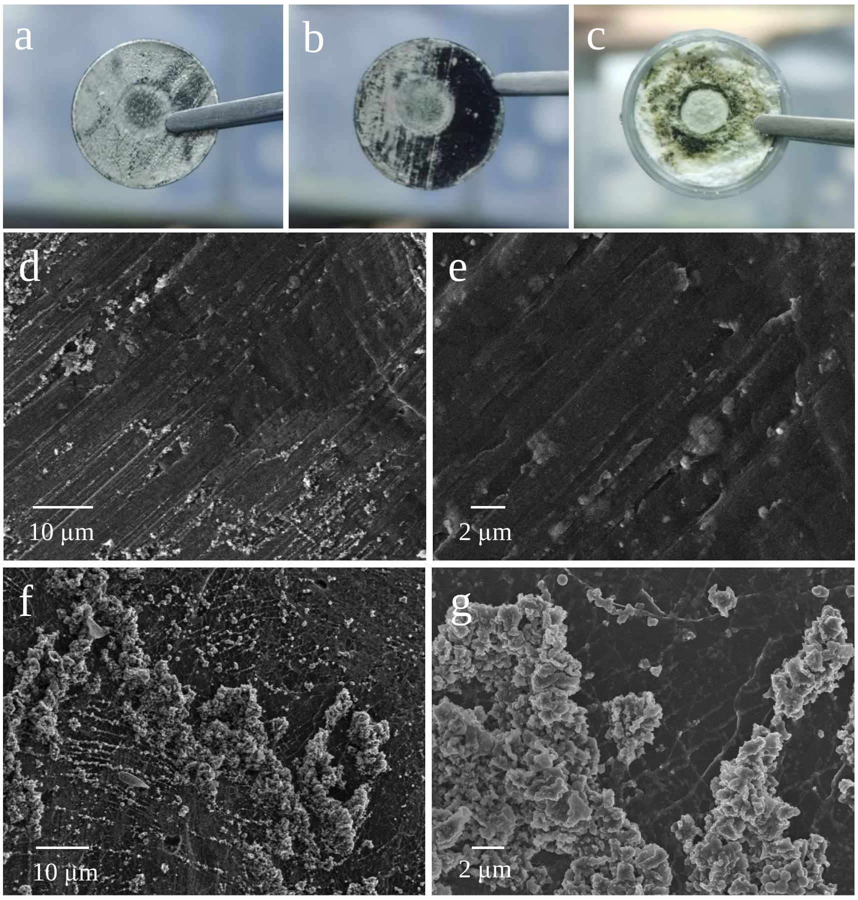

Supplementary Fig. 20 Digital photos and surface morphologies of Li anodes after cycling. **a, d, e** Li | SiO_2-SO_3Li/PVDF-HFP | O_2; **b, f, g** Li | SiO_2/PVDF-HFP | O_2; **c** Li | PVDF-HFP | O_2.

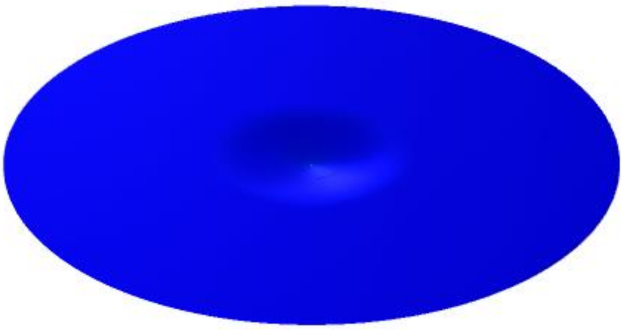

Supplementary Fig. 21 COMSOL simulation of the NNFQSE's deformation.

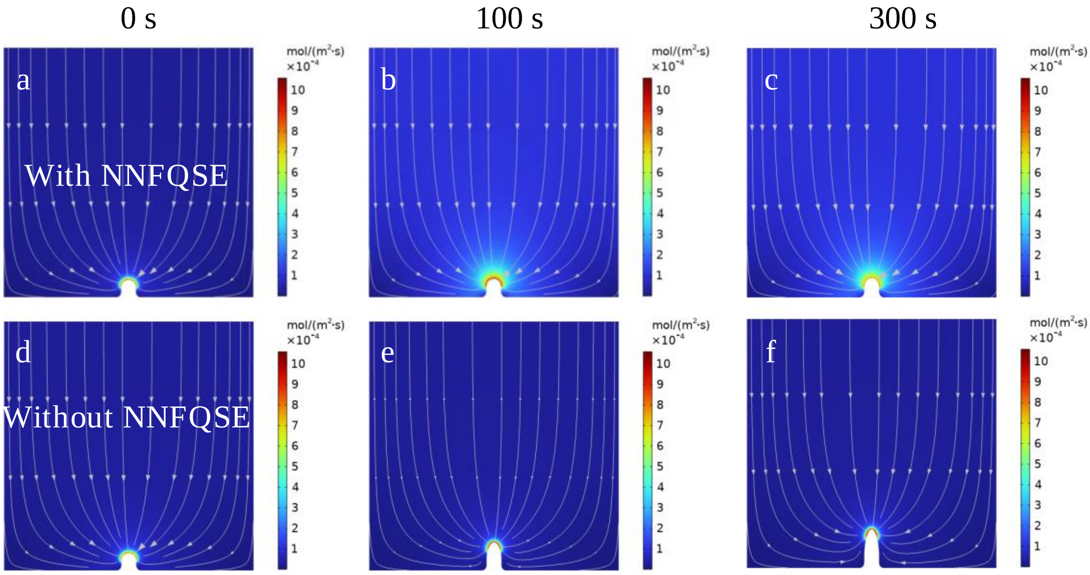

Supplementary Fig. 22. Ionic flux and Li dendrite growth simulations during prolonged time of 300 s **a-c** with NNFQSE or **d-e** without NNFQSE.

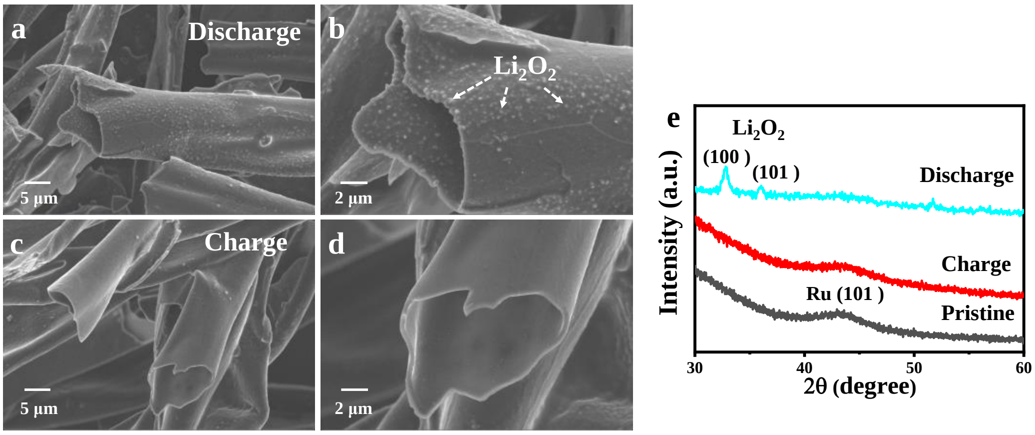

Supplementary Fig. 23 Characterization of the growth and decomposition of discharge products. a-b SEM images showing the Li_2O_2 is formed on the cathode after discharge; **c-d** SEM images showing the Li_2O_2 formed on the cathode has vanished after charge. **e** XRD analysis of cathode after discharge and charge.

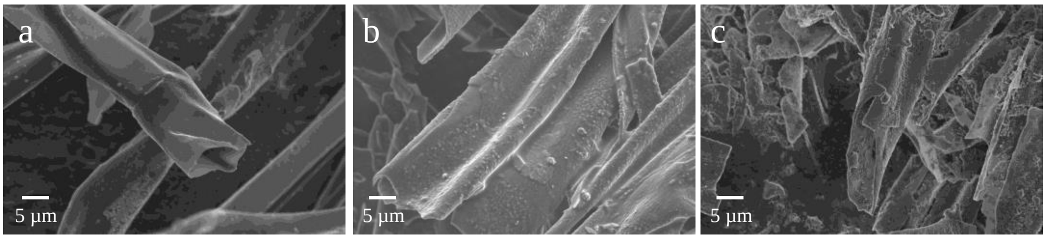

Supplementary Fig. 24 Surface morphologies of cathode after cycling. **a** Li｜SiO_2-SO_3Li/ PVDF-HFP｜O_2; **b** Li｜SiO_2/PVDF-HFP｜O_2; **c** Li｜PVDF-HFP｜O_2.

Supplementary Table 1. Porous features of PVDF-HFP, SiO_2/PVDF-HFP and SiO_2-SO_3Li/PVDF-HFP.

Electrolyte	Porosity ratio (%)	Volume density (g ml^{-1})	Apparent (skeleton) density (g ml^{-1})	Total pore volume (mL g^{-1})	Total pore area (m^2 g^{-1})
PVDF-HFP	74.6	0.34	1.34	2.19	7.57
SiO_2 /PVDF-HFP	58.4	0.59	1.43	0.98	16.06
SiO_2-SO_3Li /PVDF-HFP	62.9	0.48	1.28	1.32	18.27

Supplementary References

1. Xu, J. *et al.* Towards High Performance Li-S Batteries via Sulfonate‐Rich COF ‐ Modified Separator. *Adv. Mater.* **33**, 2105178, doi:10.1002/adma.202105178 (2021).
2. Cao, Y. *et al.* Ion Selective Covalent Organic Framework Enabling Enhanced Electrochemical Performance of Lithium−Sulfur Batteries. *Nano Lett.* **21**, 2997−3006, doi:10.1021/acs.nanolett.1c00163 (2021).
3. Jeong, K. *et al.* Solvent-Free, Single Lithium-Ion Conducting Covalent Organic Frameworks. *J. Am. Chem. Soc.* **141**, 5880−5885, doi:10.1021/jacs.9b00543 (2019).
4. Qiao, L. X. *et al.* Stable non-corrosive sulfonimide salt for 4-V-class lithium metal batteries. *Nat. Mater.* **21**, 455–462, doi:10.1038/s41563-021-01190-1 (2022).
5. Song, H. *et al.* Hierarchically Porous, Ultrathick, "Breathable" Wood‐Derived Cathode for Lithium‐Oxygen Batteries. *Advanced Energy Materials* **8**, 1701203 (2018).
6. Wu, Z. Z. *et al.* Evolving aprotic Li–air batteries. *Chem. Soc. Rev.* **51**, 8045, doi:10.1039/D2CS00003B (2022).
